# Supplementary material for: Tunable positions of Weyl nodes via magnetism and pressure in the ferromagnetic Weyl semimetal CeAlSi
Source: Nat Commun. 2024 Feb 17;15:1467. doi: 10.1038/s41467-024-45658-5 (PMC10874455; doi:10.1038/s41467-024-45658-5)
Supplement: Supplementary file 1 — Supplementary Information [file 41467_2024_45658_MOESM1_ESM.pdf]

Supplementary Information for

Tunable Weyl nodes via magnetism and pressure in the ferromagnetic Weyl semimetal CeAlSi

Supplementary Note 1: Experimental details

Electrical, thermoelectrical transport, and thermodynamic measurements. For transport measurements, a single crystal was cut into a bar shape. A standard six-probe method was used for the longitudinal resistivity and transverse Hall measurements. For thermoelectrical transport measurements, the Seebeck and Nernst signal were measured simultaneously, and the temperature gradient (ΔT) was determined by a differential AuFe/chromel-P thermocouple which had been calibrated carefully in magnetic fields. The cold end of the thermocouple was directly connected to the heat sink, and the temperature of the cold end was the same as the base temperature (T_B) which was measured by a Cernox thermometer. The temperature of the sample (T_s), i.e., the T used in Figs. 2 and 3, was determined to be the average of the cold and the hot ends, i.e., $T_s = T_B + \Delta T/2$. Electrical transport data were collected in a physical property measurement system (PPMS, Quantum Design), and thermoelectrical transport data were collected in a home-built ^{4}He cryostat. Magnetic susceptibility and specific heat measurements were performed in a magnetic property measurement system (MPMS, Quantum Design) and a PPMS, respectively.

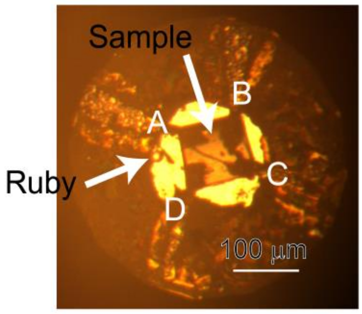

Supplementary Figure 1 | A picture of the sample chamber for high-pressure electrical transport measurement. The dimensions of the sample are ~ 70 × 70 × 15 μm^3.

For electrical transport measurements under high pressure, a van der Pauw method was used, as shown in Supplementary Fig. 1. The single crystal with a dimension of ~ 70 μm × 70 μm × 15 μm was cut by a focused ion beam (FIB) along the c axis, and the surface of the single crystal is the ab plane. Magnetic field was applied perpendicular to the plane. For the calculation of resistivity, the following equation is adopted[1],

$$\rho = \frac{\pi d}{\ln 2}\left(\frac{R_{AB}+R_{AD}}{2}\right)\cdot f\left(\frac{R_{AB}}{R_{AD}}\right),$$

where the function $f(x)$ satisfies the equation,

$$\exp\left(-\frac{\ln 2}{f(x)}\right)\cdot\cosh\left[\left(\frac{x-1}{x+1}\right)\frac{\ln 2}{f(x)}\right]=1/2,$$

d is the thickness of the sample, R_{AB} and R_{AD} are the resistance of the sample along different directions, as shown in Supplementary Fig. 1. Given the square shape of the sample and the tetragonal structure of CeAlSi, R_{AB}/R_{AD} is assumed to be ~ 1. For $x < 2.2$, $f(x) \approx 1/\cosh(\ln(x)/2.403)$ with an error of less than 0.1%.

Angle-resolved photoemission spectroscopy (ARPES) measurements. ARPES measurements were performed using the 1^3-ARPES end station of the UE-112-PGM2 beamline at the Helmholtz Zentrum Berlin BESSY-II light source. The energy and angular resolutions were set to better than 5 meV and 0.1°, respectively. Samples were cleaved *in situ*, yielding flat and mirrorlike (001) surfaces. During the experiments, the vacuum conditions were maintained better than 6×10^{-11} Torr. We used linearly horizontal polarized photons for the resonant and core-level photoemission measurements, and left-handed circularly (CR^{+}) polarized photons for the near-E_F band structure measurements.

Synchrotron XRD measurements under pressure. High-pressure angle-dispersive XRD (wavelength: 0.434 Å) measurements of ground CeAlSi powder were performed at beamline 13-BMC of the Advanced Photon Source, Argonne National Laboratory. The powder of CeAlSi was loaded into a sample chamber sealed by a rhenium gasket. A symmetric diamond anvil cell (DAC) was used to generate quasi-hydrostatic pressure using silicone oil as the pressure-transmitting medium. The pressure inside the sample chamber was determined by the shift of ruby fluorescence[2]. Experimental parameters between the sample and detector were calibrated using the standard LaB_6. All two-dimensional XRD images were analyzed using Dioptas[3], yielding one-dimensional intensity versus diffraction angle patterns. Rietveld analyses were performed by using the general structure and analysis system (GSAS) software[4].

High-pressure *ac* magnetic susceptibility measurements. The mutual induction method was used for the *ac* magnetic susceptibility measurements. The sample together with a piece of Pb were put inside the handmade primary/secondary coils of about 50 turns for each. The primary coil is driven by ac current of 1 mA and 317.7 Hz, while the output signal from the secondary coil was measured with a lock-in amplifier Stanford SR 830. The glycerol is employed as the pressure transmitting medium for the high-pressure measurements in cubic-anvil cell (CAC), and the pressure values were estimated based on the superconducting transition of Pb at low temperatures.

Density functional theory (DFT) calculations. First-principles calculations were carried out by using the Vienna *ab initio* Simulation Package (VASP)[5,6]. Exchange-correlation effects were treated by using a Perdew-Burke-Ernzerhof (PBE)-type generalized gradient approximation (GGA)[7,8] with the projector-augmented-wave (PAW) potential[9,10]. An on-site Coulomb interaction was added for Ce *f*-electrons within the GGA+U scheme with U_{eff} = 6 eV. The cutoff energy of the plane-wave basis was fixed at 500 eV. A 15×15×15 Γ-centered k mesh based on the Monkhorst-Pack method was selected to sample the Brillouin zone. The energy and force difference criteria were defined as 10^{-6} eV and 0.01 eV/Å for self-consistent convergence. To simulate paramagnetic CeAlSi, we treated the $4f$ electrons on Ce as core electrons. Spin-orbit coupling (SOC) was considered in a self-consistent manner. The WANNIER90 package[11,12] was adopted to construct Wannier functions from the first-principles results

without an iterative maximal-localization procedure[13]. The WANNIERTOOLS code[14] was used to find Weyl points. For the calculations for the pressure evolution of magnetic moments, the full-potential augmented plane-wave and local orbital methods, as implemented in the WIEN2k code, was adopted[15]. The PBE-type GGA was used for the exchange-correlation functional. The $R_{MT}K_{MAX}$ were set to be 8.0 and we used 1000 k-point meshes for the whole Brillouin zone with U_{eff} = 6 eV for Ce with turning on SOC.

Supplementary Note 2: Basic preoperties of CeAlSi at ambient pressure

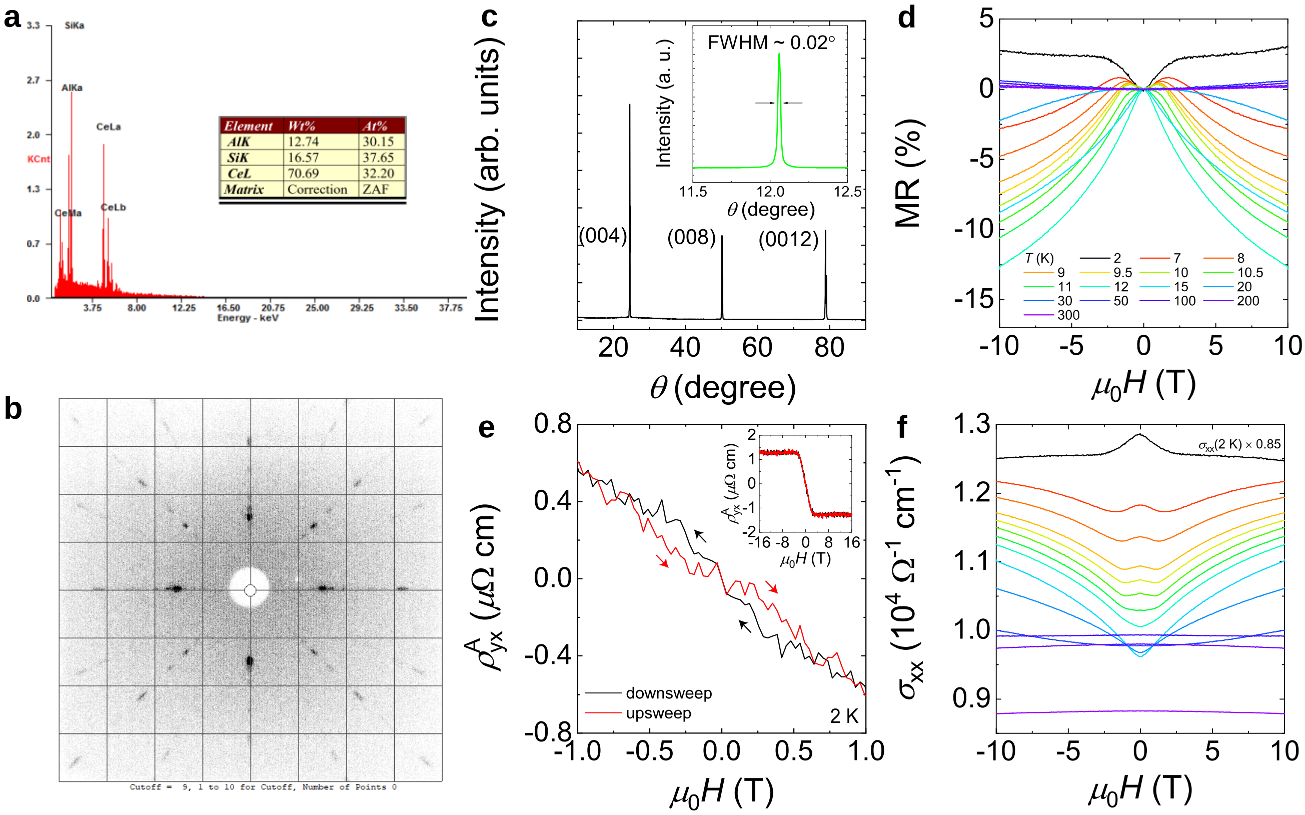

Supplementary Figure 2 | Basic preoperties of CeAlSi at ambient pressure. a X-ray diffraction (XRD) pattern of the as-grown CeAlSi single crystals. a Compositional analysis of the as-grown single crystals. **b** Laue diffraction pattern of the (001) plane. **c** XRD pattern from the largest natural surface of a CeAlSi single crystal. The largest natural surface is the *ab* plane. Inset shows the rocking curves of the (004) peaks for CeAlSi. The FWHM (full width at half maximum) of CeAlSi is 0.02°, and the small values of FWHM indicates the high quality of the as-grown single crystals. **b** Magnetoresistance (MR) [MR = $\Delta\rho_{xx}(H)/\rho_{xx}(0)\times100\%$] of CeAlSi at different temperatures. **c** The loop-shaped Hall effect (LHE) in CeAlSi. Inset displays the anomalous Hall resistivity (ρ_{yx}^{A}) at 2 K after subtracting the ordinary contribution through $\rho_{yx} = R_0B + \rho_{yx}^{A}$. **d** Longitudinal conductivity at different temperatures.

Supplementary Note 3: Anomalous transport and analysis in CeAlSi

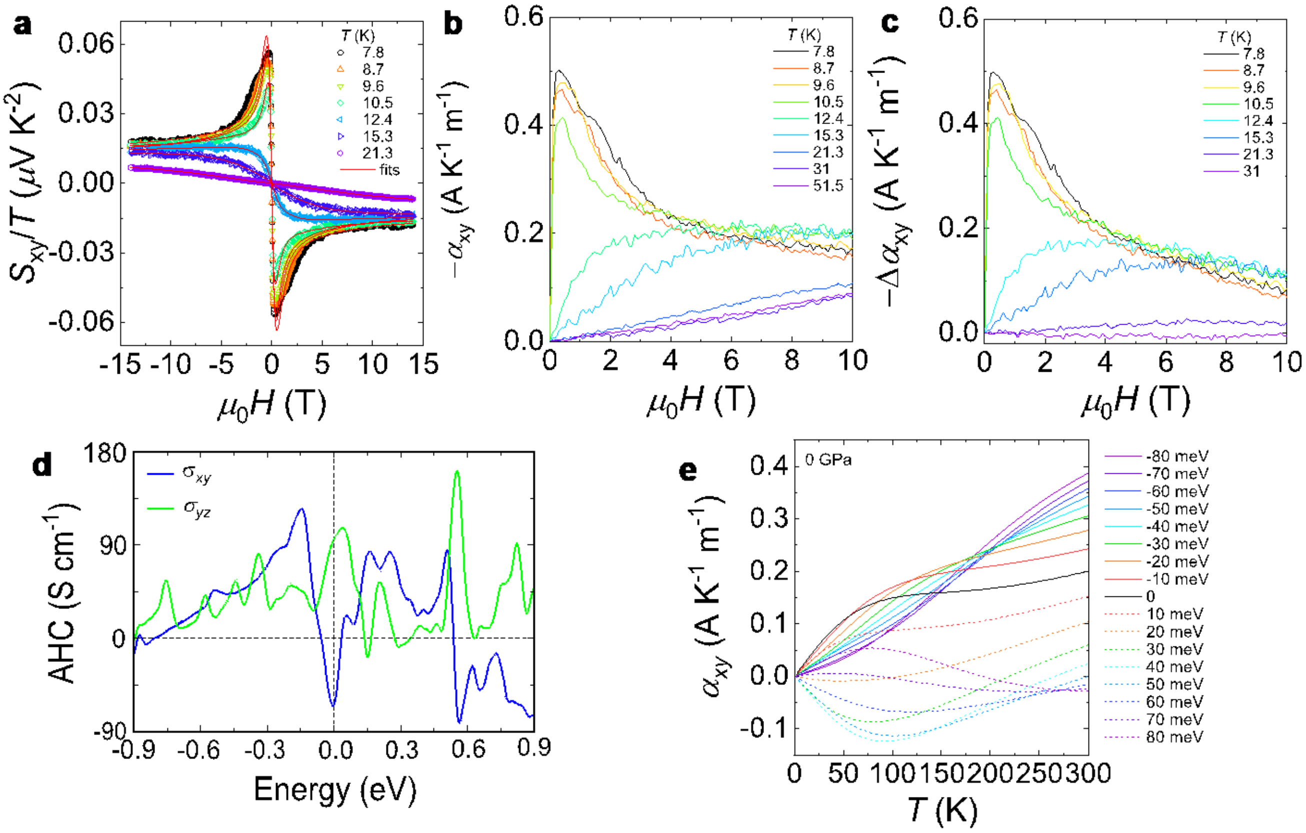

Supplementary Figure 3 | Anomalous transport and analysis in CeAlSi. a Fits to the Nernst signal normalized to the temperature at selected temperatures. **b** Nernst conductivity ($-\Delta\alpha_{xy}$) as a function of field at several selected temperatures. The Nernst conductivities for 31 and 51.5 K nearly overlap. To obtain the anomalous contributions, the data at 51.5 K is taken as the ordinary contribution to be deducted. **c** Anomalous Nernst conductivity ($-\Delta\alpha_{xy}$) as a function of field at several selected temperatures, $-\Delta\alpha_{xy} = -[\alpha_{xy}(T) - \alpha_{xy}(51.5\text{ K})]$. **d** Calculated anomalous conductivity (AHC) with a 200×200×200 Γ-centered k mesh. The calculated AHC is consistent with that in Fig. 1(g). **e** Temperature dependence of anomalous Nernst conductivity (ANC) at different chemical potentials. The obtained AHC shown in **c** is slightly larger than the theoretically estimated.

Supplementary Note 4: In-plane anomalous Hall effect and anomalous Nernst effect in CeAlSi at ambient pressure

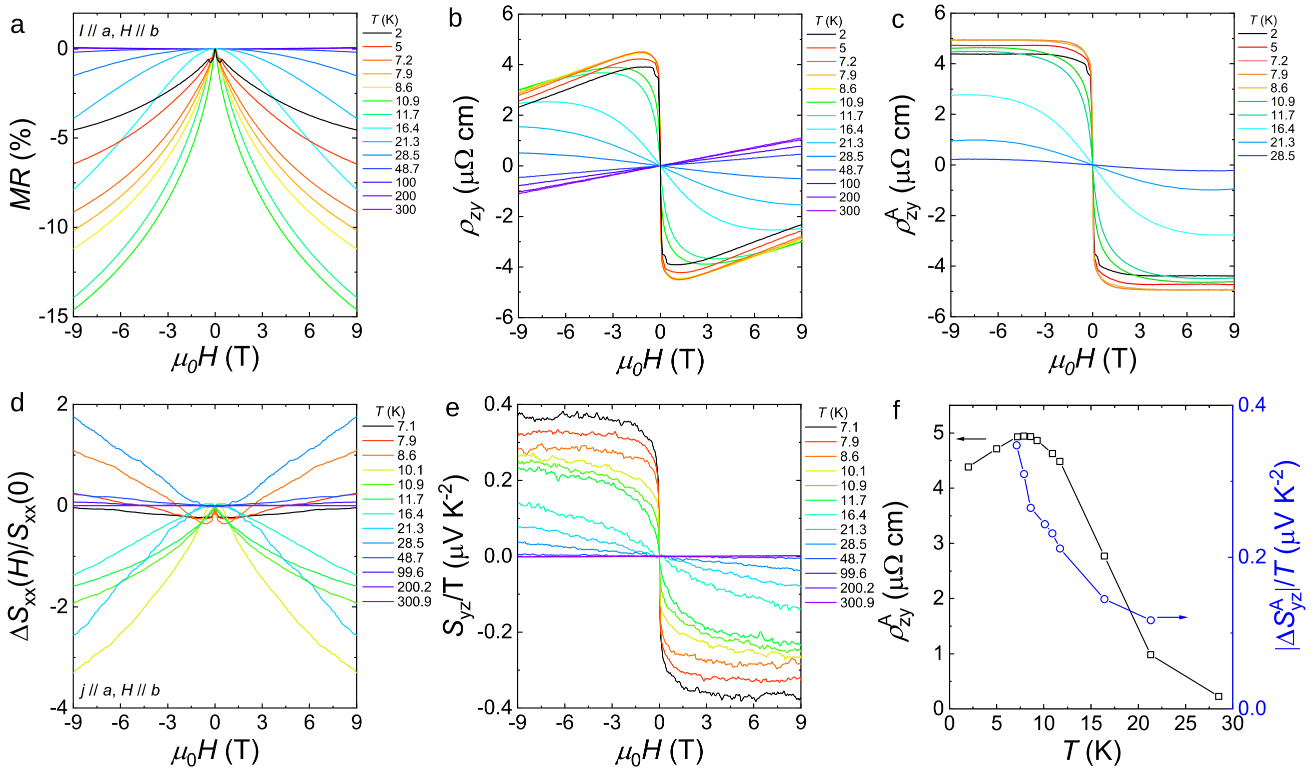

Supplementary Figure 4 | Anomalous Hall/Nernst effects of CeAlSi with in-plane magnetic field. **a** In-plane magnetoresistance (MR). **b** Hall resistivity. **c** Anomalous Hall resistivity after subtracting a linear background. **d** Normalized magneto-Seebeck signal at different temperatures with the magnetic field applied along the *b* axis. **e** Nernst signal normalized to the temperature at different temperatures. **f** Tempeature-dependent anomalous Hall resistivity and the amplitude of anomalous Nernst signal normalized to the temperature ($|S^{A}_{yz}|/T$).

To gain insight into the anisotropic characteristics of CeAlSi, we conducted electrical and thermoelectrical transport measurements while applying an in-plane magnetic field. In Supplementary Fig. 4(a), the in-plane magnetoresistance (MR) is presented at various temperatures, and notably, it exhibits a negative behavior at lower temperatures, albeit distinct from the observations in Supplementary Fig. 2(b). Supplementary Figs. 4(b) and 4(c) display the in-plane Hall resistivity and anomalous Hall resistivity, respectively. These results closely mirror what has been observed when a magnetic field is applied along the *c* axis. Furthermore, in Supplementary Fig. 4(d), we showcase the normalized magneto-Seebeck signal at different temperatures. In comparison with the data in Fig. 3(b), the Seebeck signal exhibits a more intricate behavior, which is attributed to the in-plane noncollinear ferromagnetic configuration. Supplementary Fig. 4(e) reveals the normalized Nernst signal in relation to temperature, and upon fitting this data using Eq. (1), we derive the amplitude of the anomalous Nernst signal normalized to temperature. These profiles are illustrated in Supplementary Fig. 4(f). It is noteworthy that $|S^{A}_{yz}|/T$ increases with decreasing temperature. Below T_C, the curve's slope rises, indicating the enhancement of the anomalous Nernst effect through ferromagnetic coupling.

Supplementary Note 5: Universal scaling relation between the anomalous Hall conductivity and the longitudinal conductivity in CeAlSi.

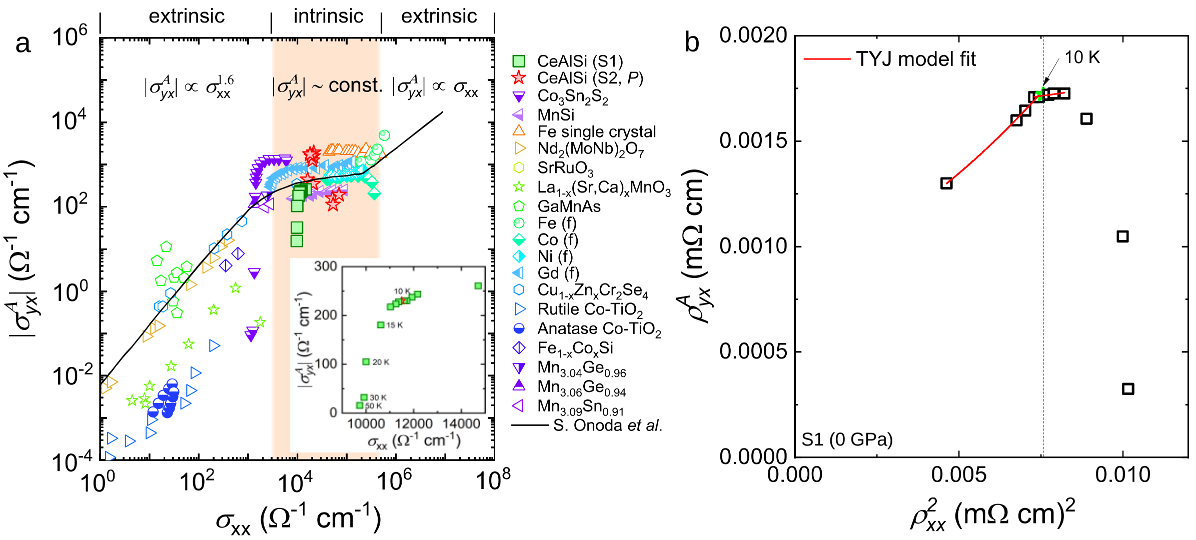

Supplementary Figure 5 | Intrinsic anomalous Hall effect in CeAlSi. (a) Absolute value of anomalous Hall conductivity $|\sigma^A_{xy}|$ as a function of longitudinal conductivity σ_{xx} of CeAlSi under ambient and high pressure. For better comparison, several pure metals (Fe, Co, Ni, Gd)[16], oxides [$Nd_2(MoNb)_2O_7$, $La_{1-x}(Sr,Ca)_xMnO_3$, $SrRuO_3$][17], chalcogenide spinels ($Cu_{1-x}Zn_xCr_2Se_4$)[18], magnetic semiconductors (GaMnAs, anatase–Co–TiO_2, rutile–Co–TiO_2)[17], $Co_3Sn_2S_2$ [19], MnSi[20], $Fe_{1-x}Co_xSi$[20], Mn_3Ge[21], and Mn_3Sn[22] have been plotted together. The solid lines in three regimes represent $|\sigma^A_{xy}| \propto \sigma^{1.6}_{xx}$, $|\sigma^A_{xy}| \sim$ const., and $|\sigma^A_{xy}| \propto \sigma_{xx}$, for the dirty, intermediate, and clean regimes, respectively[16,22]. Inset shows the data in conventional coordinates. (**b**) ρ^A_{yx} as a function of ρ^2_{xx} for S1 at ambient pressure, and the fitting by using the Tian-Ye-Jin (TYJ) model, $\rho^A_{yx} = a\rho_{xx0} + b\rho^2_{xx}$, where the first and the second terms on the right-hand side of the equation represent the extrinsic and intrinsic contributions, respectively[23]. The data point at 10 K is marked out, below which the system is in the ferromagnetic state.

In the case of CeAlSi, whether at ambient (T > 15 K) or high pressures, the anomalous Hall conductivity consistently falls within the intermediate regime, indicating an intrinsic origin of the anomalous Hall effect. Notably, for S1, we have provided temperature-dependent data, whereas for S2, pressure-dependent data at 2 K is presented. To delve deeper into the analysis of the intrinsic contribution to the anomalous Hall effect, we have applied the TYJ model to fit the data, as demonstrated in Supplementary Fig. 5(b). It is evident that two distinct regimes can be well-fitted: one corresponds to the ferromagnetic state, and the other extends across the ferromagnetic transition up to 12 K. The linear correlation between ρ^A_{yx} and ρ^2_{xx} within these regimes signifies the intrinsic nature of the anomalous Hall effect. Conversely, beyond 15 K, ρ^A_{yx} $vs. \rho^2_{xx}$ deviates from a linear dependence, suggesting that the AHE may be influenced by extrinsic contributions. In a bad-metal regime [σ_{xx} <10^4 Ω^{-1}cm^{-1}], σ^A_{yx} decreases with decreasing σ_{xx} at a rate faster than linear[24]. In the case of CeAlSi, as depicted in the inset of Supplementary Fig. 5(a), the system seemingly exists within the bad-metal regime beyond 15 K, where magnetic fluctuations begin to exert dominance [as illustrated in Figs. 1(f)

and 1(k)].

Supplementary Note 6: High-pressure electrical transport results of CeAlSi

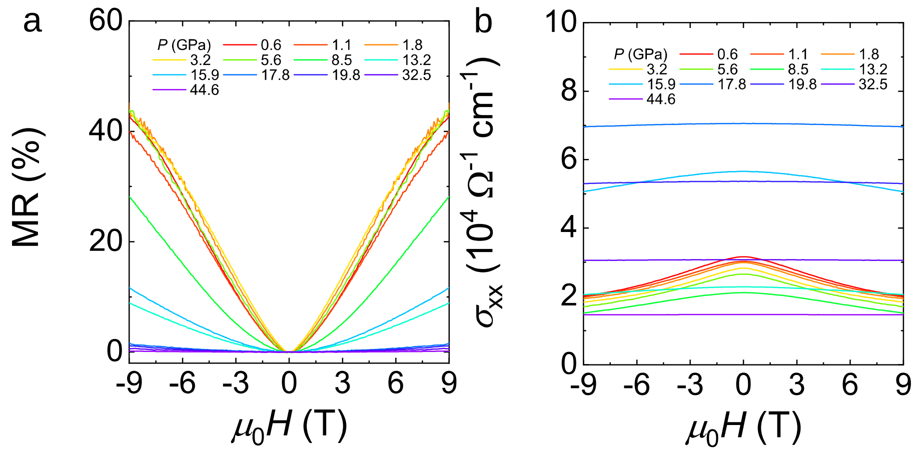

Supplementary Figure 6 | High-pressure electrical transport results of CeAlSi. a and b Magnetoresistance (MR) and conductivity as a function of field at 2 K for different pressures.

Supplementary Note 7: The pressure evolution of band structure of CeAlSi

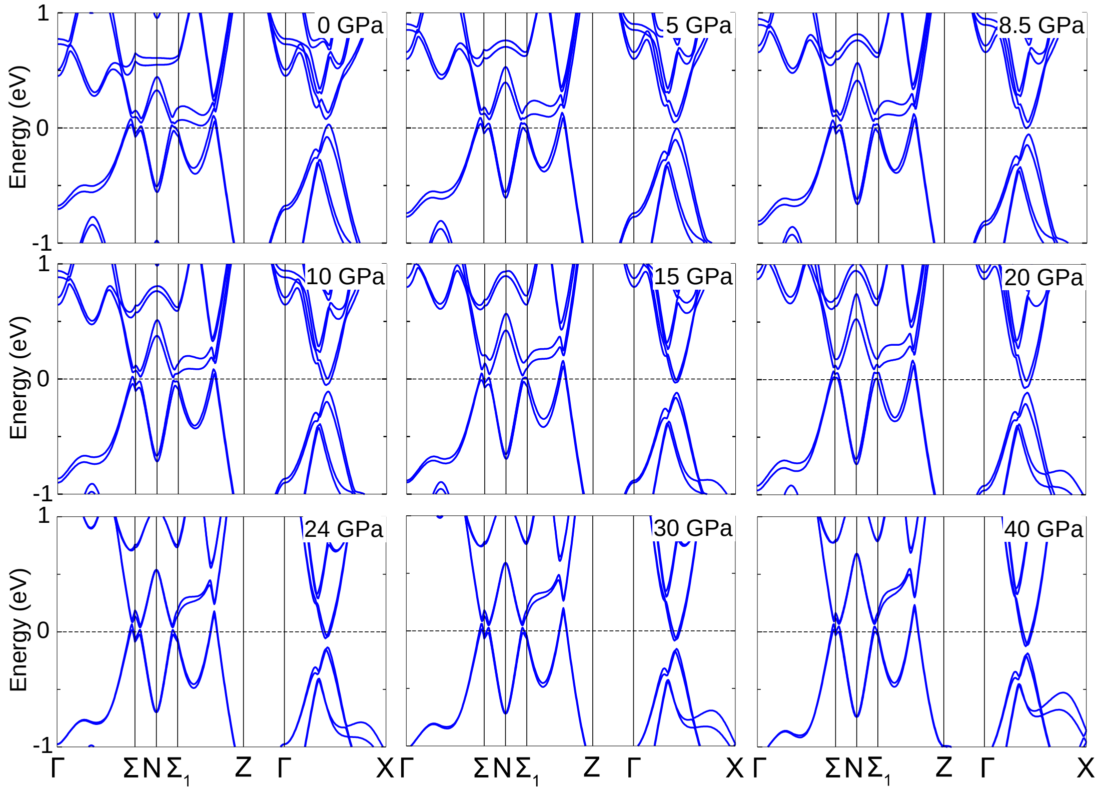

Supplementary Figure 7 | The evolution of band structure of CeAlSi under pressure with spin-orbit coupling (SOC) included. For all pressures, CeAlSi remains a Weyl semimetal, and the band structure does not change much. With increasing pressure, the hole pockets along the Γ–X line become

smaller, and then turn into electron pockets at 8.5 ~ 10 GPa, evidencing a pressure-induced Lifshitz transition.

Supplementary Note 8: The shift of Weyl nodes's positions under pressure in CeAlSi

0 GPa				
Weyl nodes	k_x	k_y	k_z	*Energy* (meV)
WP_1 (Γ-Σ)	-0.25200545	0.25237779	0.25593442	104
WP_2 (Γ-X)	-0.04079980	0.04087652	0.22217857	73
WP_3 (Γ-Σ_1)	0.22423743	0.48827042	-0.23977390	52
20 GPa				
Weyl nodes	k_x	k_y	k_z	*Energy* (meV)
WP_1 (Γ-Σ)	-0.26380646	0.26507264	0.26713738	110
WP_2 (Γ-X)	-0.03605019	0.03607966	0.22267151	-52
WP_3 (Γ-Σ_1)	0.22872763	0.45307070	-0.21025441	120

Supplementary Table 1 | Three typical Weyl nodes at ambient and 20 GPa. According to symmetry, there are 16 pairs of Weyl nodes: 4 along the Γ-Σ, 4 along Γ-X, and 8 along Γ-Σ_1. With increasing pressure, the positions of Weyl nodes are shifted by pressure, indicating that pressure could serve as an efficient parameter to tune the Weyl nodes in CeAlSi.

Supplementary Note 9: The electron correlations in pressurized CeAlSi

In Supplementary Fig. 8(a), we present the low-temperature resistivity data, with the critical temperatures (T_Cs) determined from *ac* susceptibility measurements marked for reference. It's evident that, beyond 5.6 GPa, the low-temperature resistivity exhibits distinct behavior. At 8.5 GPa, an additional broad peak emerges (indicated by a black arrow), and this profile bears a resemblance to observations in certain Ce-based heavy fermion systems characterized by valence fluctuations[25-27]. In Supplementary Fig. 8(b), we present semilogarithmic plots of the resistivity above 13.2 GPa. At pressures of 15.9, 17.9, and 19.8 GPa, the resistivity initially decreases to a minimum and then exhibits a characteristic -*lnT* dependence, typical of Kondo systems[25]. Below approximately 5 K, the deviation from a logarithmic increase suggests that the spin-compensated state or the influence of Ruderman-

Kittel-Kasuya-Yosida (RKKY) interactions between magnetic impurities may come into play[29,30].

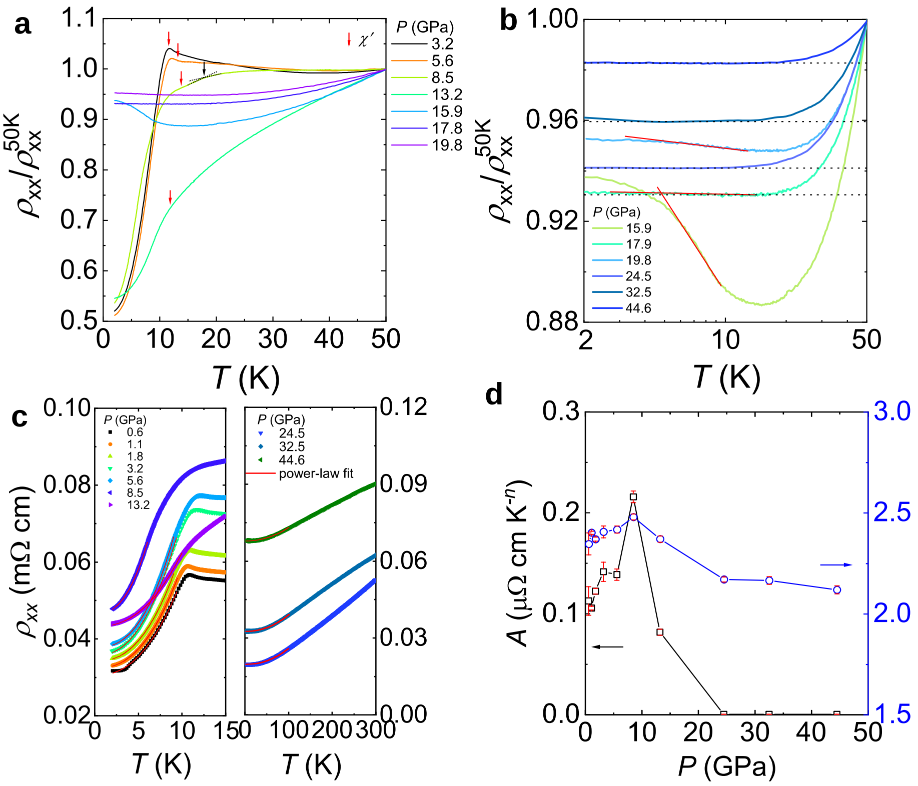

Supplementary Figure 8 | a Low-temperature resistivity normalized to the data at 50 K. Red arrows represent the evolution of ferromagnetic transition with pressure deduced from *ac* susceptibility. The black arrow for 8.5 GPa indicates another peak in resistivity. **b** Semilogarithmic plots of the temperature dependence of resistivity at several selected pressures. Red solid line is a guide to the eye and shows a -*lnT* dependence, which is maybe due to a contribution from the conduction electron-magnetic impurity interaction[25]. **c** Longitudinal resistivity at various pressures. The red solid lines represent the fitting achieved through a power law, namely, $\rho = \rho_0 + AT^n$. In left panel, the data was fitted within the magnetic ordering state, whereas in right panel, the fitting encompassed data below 100 K, attributed to the display of typical metallic behavior in resistivity. Notably, data points at 15.9, 17.8 and 19.8 GPa were omitted from the fitting process due to the resistivity's divergence at low temperatures. **d** The yielded parameters by fitting the low-temperature resistivit. Error bars are deduced from the fit.

To further address the electron correlation effect, we fitted the low-temperature data at different pressures, using $\rho = \rho_0 + AT^n$, as shown in Supplementary Fig. 8(c). The yielded parameters are displayed in Supplementary Fig. 8(d). Here, the ρ_0, A, and n represent the residual resistivity, the inelastic electron-electron scattering coefficient, and the exponentiation of temperature, respectively. For a Fermi-liquid system, n is 2. The inelastic electron-electron scattering is enormously stronger (by three or four orders of magnitude) in heavy-fermion materials than it is in normal metals[31]. Typically, it is governed by the density of states at the Fermi energy and a simple relation $\gamma_N \sim \sqrt{A}$ is fairly well obeyed across the family of compounds[31]. From the fit, one can see that the n we obtained is slightly larger than 2, but far lower than 5 that from electron-phonon scattering in the low-temperature limit. Hence, CeAlSi is presumed to exhibit Fermi-liquid behavior within both lower and higher pressure

ranges. Notably, we refrained from fitting the data spanning 15.9 to 19.8 GPa due to the observed divergence of resistivity as temperature decreases. However, strikingly, the inelastic electron-electron scattering coefficient *A* displays a nonmonotonic evolution with pressure. It initially increases, and then peaks at ~ 8.5 GPa with a value of 0.216 ± 0.006 $\mu\Omega$ cm K^{-n}. This value is comparable with those in heavy-fermion systems, for example, 0.55 ± 0.05 $\mu\Omega$ cm K^{-2} in the typical heavy fermion superconductor UPt_3 for a current parallel to the hexagonal *c* axis[31]. Under higher pressure, the *A* decreases down to 0.00028 ± 0.00002 $\mu\Omega$ cm K^{-n} (24.5 GPa), three orders of magnitude smaller than that at 8.5 GPa, which means that higher pressures drive CeAlSi into a normal metal. The pressure evolution of *A* and *n* is in line with the magnetic transition under pressure, and it may be attributed to the effect of charge carriers scattering on ferromagnetic fluctuations near the transition. Nevertheless, the pressure-induced variations in *A* values cannot be entirely attributed to the scattering mechanism alone. We maintain the stance that electron-electron correlations might also be influenced by pressure to some extent.

In addition, according to the Doniach phase diagram, the Kondo interaction and the RKKY interaction compete with each other[32], and the balance between them would lead to a quantum critical point. However, this is not observed in CeAlSi. Based on the periodic Anderson model, the valence fluctuations can no longer be neglected[26]. With continues tuning (herein, i.e., pressure), the system may be driven into a mixed valence regime, and then a normal metal regime[33], which is likely in line with what we observed in CeAlSi.

Supplementary References

1. Ramadan, A. A., Gould, R. D. & Ashour, A. On the Van der Pauw method of resistivity measurements. *Thin Solid Films* **239**, 272–275 (1994).
2. Mao, H. K., Xu, J. & Bell, P. M. Calibration of the ruby pressure gauge to 800 kbar under quasi-hydrostatic conditions. *J. Geophys. Res.* **91**, 4673 (1986).
3. Prescher, C. & Prakapenka, V. B. *DIOPTAS*: a program for reduction of two-dimensional X-ray diffraction data and data exploration. *High Press. Res.* **35**, 223–230 (2015).
4. Larson, A. C., & Von Dreele, R. B. Gsas. *Report lAUR*, 86–748 (1994).
5. Kresse, G. & Furthmüller, J. Efficiency of ab-initio total energy calculations for metals and semiconductors using a plane-wave basis set. *Comput. Mater. Sci.* **6**, 15–50 (1996).
6. Kresse, G. & Furthmüller, J. Efficient iterative schemes for *ab initio* total-energy calculations using a plane-wave basis set. *Phys. Rev. B* **54**, 11169–11186 (1996).
7. Perdew, J. P., Burke, K. & Ernzerhof, M. Generalized gradient approximation made simple. *Phys. Rev. Lett.* **77**, 3865–3868 (1996).
8. Perdew, J. P. & Wang, Y. Accurate and simple analytic representation of the electron-gas correlation energy. *Phys. Rev. B* **45**, 13244–13249 (1992).
9. Lehtomäki, J., Makkonen, I., Caro, M. A., Harju, A. & Lopez-Acevedo, O. Orbital-free density functional theory implementation with the projector augmented-wave method. *J. Chem. Phys.* **141**, 234102 (2014).
10. Blöchl, P. E. Projector augmented-wave method. *Phys. Rev. B* **50**, 17953–17979 (1994).
11. Souza, I., Marzari, N. & Vanderbilt, D. Maximally localized Wannier functions for entangled energy bands. *Phys. Rev. B* **65**, 035109 (2001).
12. Mostofi, A. A. *et al.* wannier90: A tool for obtaining maximally-localized Wannier functions.

Comput. Phys. Commun. **178**, 685–699 (2008).
13. Marzari, N. & Vanderbilt, D. Maximally localized generalized Wannier functions for composite energy bands. *Phys. Rev. B* **56**, 12847–12865 (1997).
14. Wu, Q., Zhang, S., Song, H.-F., Troyer, M. & Soluyanov, A. A. WannierTools: An open-source software package for novel topological materials. *Comput. Phys. Commun.* **224**, 405–416 (2018).
15. Blaha, P., Schwarz, K., Madsen, G. K. H., Kvasnicka, D. & Luitz, J. Wien2k: An augmented plane wave plus local orbital program for calculating the crystal properties (Technical University of Wien in Austria, ISBN39501031-1-2) (2001).
16. Miyasato, T. *et al.* Crossover behavior of the anomalous Hall effect and anomalous Nernst effect in itinerant ferromagnets. *Phys. Rev. Lett.* **99**, 086602 (2007).
17. Onoda, S., Sugimoto, N. & Nagaosa, N. Quantum transport theory of anomalous electric, thermoelectric, and thermal Hall effects in ferromagnets. *Phys. Rev. B* **77**, 165103 (2008).
18. Lee, W.-L., Watauchi, S., Miller, V. L., Cava, R. J. & Ong, N. P. Anomalous Hall heat current and Nernst effect in the $CuCr_2Se_{4-x}Br_x$ ferromagnet. *Phys. Rev. Lett.* **93**, 226601 (2004).
19. Liu, E. *et al.* Giant anomalous Hall effect in a ferromagnetic kagome-lattice semimetal. *Nat. Phys.* **14**, 1125–1131 (2018).
20. Manyala, N. *et al.* Large anomalous Hall effect in a silicon-based magnetic semiconductor. *Nat. Mater.* **3**, 255–262 (2004).
21. Chen, T. *et al.* Anomalous transport due to Weyl fermions in the chiral antiferromagnets Mn_3X, X = Sn, Ge. *Nat. Commun.* **12**, 572 (2021).
22. Onoda, S., Sugimoto, N. & Nagaosa, N. Intrinsic versus extrinsic anomalous Hall effect in ferromagnets. *Phys. Rev. Lett.* **97**, 126602 (2006).
23. Shen, J. *et al.*, Intrinsically enhanced anomalous Hall conductivity and Hall angle in Sb-doped magnetic Weyl semimetal $Co_3Sn_2S_2$. *APL Mater.* **10**, 090705 (2022).
24. Nagaosa, N., Sinova, J., Onoda, S., MacDonald, A. H. & Ong, N. P. Anomalous Hall effect. *Rev. Mod. Phys.* **82**, 1539–1592 (2010).
25. Kondo, J. Resistance minimum in dilute magnetic alloys. *Prog. Theo. Phys.* **32**, 37–49 (1964).
26. Weng, Z. F. *et al.* Multiple quantum phase transitions and superconductivity in Ce-based heavy fermions. *Rep. Prog. Phys.* **79**, 094503 (2016).
27. Ren, Z. *et al.*, Giant overlap between the magnetic and superconducting phases of under pressure. *Phys. Rev. X* **4**, 031055(2014).
28. Scheerer, G. W. *et al.*, The dominant role of critical valence fluctuations on high *Tc* superconductivity in heavy fermions. *npj Quantum Mater.* **3**, 41 (2018).
29. Liang, V. K. C. & Tsuei, C. C. Kondo effect in an amorphous $Ni_{41}Pd_{41}B_{18}$ alloy containing Cr. *Phys. Rev. B* **7**, 3215 (1973).
30. Ding, X. X. *et al.*, Crossover from Kondo to Fermi-liquid behavior induced by high magnetic field in 1*T*-VTe_2 single crystals. *Phys. Rev. B* **103**, 125115 (2021).
31. Joynt, R. & Taillefer, L. The superconducting phases of UPt_3. *Rev. Mod. Phys.* **74**, 235 (2002).
32. Doniach, S. The Kondo lattice and weak antiferromagnetism. *Physica B+ C* **91**, 231-234 (1977).
33. Khomskii, D. I. Transition metal compounds. 501 (2014).
